# Dysregulated B Cell Expression of RANKL and OPG Correlates with Loss of Bone Mineral Density in HIV Infection

**DOI:** 10.1371/journal.ppat.1004497

**Published:** 2014-11-13

**Authors:** Kehmia Titanji, Aswani Vunnava, Anandi N. Sheth, Cecile Delille, Jeffrey L. Lennox, Sara E. Sanford, Antonina Foster, Andrea Knezevic, Kirk A. Easley, M. Neale Weitzmann, Ighovwerha Ofotokun

**Affiliations:** 1 Division of Endocrinology, Metabolism and Lipids, Emory University School of Medicine, Atlanta, Georgia, United States of America; 2 Division of Infectious Diseases, Department of Medicine, Emory University School of Medicine, Atlanta, Georgia, United States of America; 3 Department of Biostatistics and Bioinformatics, Rollins School of Public Health, Emory University, Atlanta, Georgia, United States of America; 4 Atlanta VA Medical Center, Decatur, Georgia, United States of America; 5 Winship Cancer Institute, Emory University, Atlanta, Georgia, United States of America; Vaccine Research Center, United States of America

## Abstract

HIV infection is associated with high rates of osteopenia and osteoporosis, but the mechanisms involved are unclear. We recently reported that bone loss in the HIV transgenic rat model was associated with upregulation of B cell expression of the key osteoclastogenic cytokine receptor-activator of NF-κB ligand (RANKL), compounded by a simultaneous decline in expression of its physiological moderator, osteoprotegerin (OPG). To clinically translate these findings we performed cross-sectional immuno-skeletal profiling of HIV-uninfected and antiretroviral therapy-naïve HIV-infected individuals. Bone resorption and osteopenia were significantly higher in HIV-infected individuals. B cell expression of RANKL was significantly increased, while B cell expression of OPG was significantly diminished, conditions favoring osteoclastic bone resorption. The B cell RANKL/OPG ratio correlated significantly with total hip and femoral neck bone mineral density (BMD), T- and/or Z-scores in HIV infected subjects, but revealed no association at the lumbar spine. B cell subset analyses revealed significant HIV-related increases in RANKL-expressing naïve, resting memory and exhausted tissue-like memory B cells. By contrast, the net B cell OPG decrease in HIV-infected individuals resulted from a significant decline in resting memory B cells, a population containing a high frequency of OPG-expressing cells, concurrent with a significant increase in exhausted tissue-like memory B cells, a population with a lower frequency of OPG-expressing cells. These data validate our pre-clinical findings of an immuno-centric mechanism for accelerated HIV-induced bone loss, aligned with B cell dysfunction.

## Author Summary

HIV infection causes significant bone loss and skeletal deterioration, leading to fractures that are often devastating and incur significant financial burden on patients and their families. HIV-infected individuals have up to a five-fold higher risk of bone fractures, and the increasing average age of people living with HIV/AIDS has triggered fears of an impending epidemic of bone fractures in this population. Antiretroviral therapy, used to manage HIV infection, fails to prevent, but rather paradoxically accelerates skeletal decline. The underlying mechanisms of HIV-induced bone loss are poorly understood. The aim of this study was to clarify the mechanisms of bone loss in HIV-infected patients, in an effort to better understand how bone loss and fractures occur, and consequently how it can be prevented in this population. The cytokine RANKL (Receptor Activator of Nuclear Factor kappa-B Ligand) helps induce bone loss. We show that RANKL expression was increased in immune cells in HIV-infected individuals. Another cytokine, osteoprotegerin (OPG), counteracts the activity of RANKL, and therefor helps prevent bone loss. OPG expression by the same immune cells was decreased in HIV-infected individuals. We conclude that disrupted immune cell expression of RANKL and OPG in HIV-infected patients contributes to bone loss.

## Introduction

The success of antiretroviral therapy (ART), a combination of drugs from multiple classes of antiviral compounds, has led to increased life expectancy among people living with HIV/AIDS. However, this increased longevity is unmasking complications of chronic HIV infection. Non-AIDS conditions including cardiovascular disease, metabolic disorders, fragility bone disease, and malignancies now account for majority of HIV related morbidity and mortality [1], [2]. For example, skeletal decline has long been recognized in this population [3], [4] and data suggest that up to 67% of HIV infected individuals are osteopenic with as many as 15% being osteoporotic [4].

Of note, ART actually inflicts an additional 2%–6% loss in bone mineral density (BMD) within the first year or two of therapy initiation [5], further worsening the already compromised skeletal status resulting from HIV infection itself [6], [7]. The underlying pathophysiology of this HIV-induced bone loss has been a subject of intense debate, fueled by the reality that co-morbidities that predispose to increased bone turnover, including renal impairment, muscle wasting, and hypogonadism are common in individuals with HIV infection, as are traditional risk factors for osteoporosis such as low body weight, smoking and excessive alcohol consumption [5], [8]–[10]. As a result of the combined effects of HIV- and ART-induced damage to the skeleton, bone fracture prevalences two- to five-fold higher than the general population have been reported in a number of large observational cohorts [11]–[16].

Mechanistically, bone loss stems from an imbalance in osteoclastic bone resorption relative to osteoblastic bone formation. Osteoclastogenic bone resorption is promoted primarily by the key osteoclastogenic cytokine, RANKL, and physiologically moderated by its decoy receptor, OPG [17], [18]. Previous work by our group and others has revealed that immune cells potently regulate physiological and pathological bone turnover. Human B cells secrete OPG [19], and we have demonstrated that under physiological conditions B lineage cells are the dominant source of OPG in the mouse bone marrow and that T cells are central regulators of B cell OPG expression through costimulatory interactions [20]. Thus lymphocytes are protective of the skeleton under basal conditions. By contrast, under inflammatory conditions both B and T cells can be considerable sources of the pro-resorptive cytokine, RANKL [21], [22]. The adaptive immune system thus potently impacts the skeleton via a convergence of immune cells and cytokine effectors, mediating critical functions in both organ systems and forming the “immuno-skeletal interface”.

Because HIV infection inflicts extensive damage to both cellular and humoral immunity [23], [24], and regulation of bone homeostasis is intricately linked to the integrity of adaptive immune responses, we hypothesized that HIV-induced bone loss may be driven in part by the disruption of the immuno-skeletal interface. In support of this notion, we recently demonstrated significant skeletal degradation and elevated osteoclastic bone resorption in the transgenic rat model of HIV infection [25]. Furthermore, the HIV transgenic rat exhibited significant decline in B cell expression of the anti-osteoclastogenic factor OPG and increased B cell expression of the pro-osteoclastogenic cytokine RANKL [25]. Because the RANKL/OPG ratio in the bone microenvironment is considered a key determinant of osteoclastogenesis, resulting imbalances in OPG and RANKL expression likely significantly contribute to increased osteoclast formation, enhanced bone resorption, and loss of BMD in this animal model of HIV infection. In the current report, we validate these pre-clinical findings in human HIV infection and show for the first time in humans a possible role for B cell dysfunction in HIV-associated bone loss. We demonstrate that B cells isolated from antiretroviral-naïve HIV-infected individuals characteristically exhibit an imbalance in B cell OPG and RANKL expression resulting in conditions favorable to enhanced osteoclastic bone resorption.

## Results

### Demographic and clinical data

Study population demographics and clinical characteristics are outlined in Table 1. Race, age, current smoking at enrollment, and reported past 30-day alcohol consumption did not significantly differ by HIV status. Sixty-nine percent of the HIV-infected group were men as compared with 48% of the HIV-negative group (P = 0.02) reflecting the sex distribution of the HIV/AIDS epidemic in our study population. A significantly greater proportion of the HIV-negative group had a history of bone fracture after 18 years of age (41.4% versus 21.0%; P = 0.02). As expected in this cohort with advanced HIV disease (average CD4 count  = 149 cells/mm^3^), body mass index (BMI) was significantly lower in the HIV-infected group (P = 0.0003).

**Table 1:** Demographic and clinical characteristics of study population.

Factor	HIV- N = 58	HIV+ N = 62	P†
	**Frequency (%)**	**Frequency (%)**	
Male Gender	28/58 (48.3%)	43/62 (69.4%)	0.02
Race‡			
- White	10/57 (17.5%)	7/62 (11.3%)	0.33
- Black	47/57 (82.5%)	55/62 (88.7%)	
Current smoking at enrollment	35/58 (60.3%)	38/62 (61.3%)	0.92
Reported alcohol consumption in past 30 days	36/58 (62.1%)	35/62 (56.5%)	0.53
History of bone fracture (after 18 years of age)	24/58 (41.4%)	13/62 (21.0%)	0.02
	***Mean (SD)***	***Mean (SD)***	
Enrollment Age (years)	41.2 (5.9)	39.2 (6.4)	0.08
Body Mass Index (kg/m^2^)	31.1 (9.0)	26.0 (5.8)	0.0003
Calcium (mg/dL)	9.3 (0.4)	9.3 (0.4)	0.81
AST/SGOT (U/L)	20.6 (7.0)	29.3 (15.3)	0.0001
ALT/SGPT (U/L)	18.1 (8.8)	27.2 (19.2)	0.001
Creatinine Clearance (mg/dL)	125.2 (43.9)	117.3 (33.1)	0.27
	***Mean (SD)***	***Mean (Q1, Q3)***	
CD4 count (cells/mm^3^)	-	149 (66, 235)	-

† Characteristics are compared between HIV serostatus groups with a two-sample t-test for continuous variables and a Chi-square test for categorical variables.

‡ One study participant was Asian and is not included in the race analysis

### Increased osteopenia in ART-naïve HIV-infected individuals

Using bone densitometry (dual energy X-ray absorptiometry, [DXA]) we quantified BMD at sites often associated with fragility fracture (left and right hip, left and right femur neck and lumbar spine), in HIV-infected individuals and uninfected controls (Table 2). T-scores and Z-scores were further derived from BMD values based on World health Organization (WHO) criteria (see methods). BMD, T-scores and Z-scores for hip and femur neck are reported as an average of left and right measurements. Mean hip BMDs were slightly diminished at all sites in HIV-infected groups compared to controls but failed to reach statistical significance. However, significantly lower mean total hip T-scores and Z-scores (P = 0.03, for both) were observed in the HIV-infected group. No difference was observed in lumber spine and femoral neck T and Z scores.

**Table 2:** BMD and bone turnover outcomes.

Outcome		HIV-		HIV+	Difference	P†
	***N***	***Mean (95% CI)***	***N***	***Mean (95% CI)***	***Mean (95% CI)***	
Total Hip BMD (g/cm^2^)*	58	1.13 (1.09, 1.18)	62	1.08 (1.04, 1.12)	−0.05 (−0.11, 0.01)	0.16
Total Hip T-Score*	58	0.69 (0.33, 1.05)	62	0.14 (−0.15, 0.44)	−0.55 (−1.01, −0.09)	0.03
Total Hip Z-Score*	58	−0.17 (−0.48, 0.14)	62	−0.64 (−0.90, −0.39)	−0.47 (−0.87, −0.08)	0.03
Femur Neck Density (g/cm^2^)*	58	1.11 (1.06, 1.15)	62	1.08 (1.04, 1.11)	−0.03 (−0.09, 0.03)	0.44
Femur Neck T-Score*	58	0.69 (0.35, 1.04)	62	0.28 (−0.02, 0.58)	−0.41 (−0.86, 0.03)	0.14
Femur Neck Z-Score*	58	−0.06 (−0.37, 0.24)	62	−0.43 (−0.68, −0.17)	−0.37 (−0.76, 0.03)	0.14
Lumbar Spine Density (g/cm^2^)	58	1.28 (1.22, 1.33)	61	1.27 (1.23, 1.31)	−0.01 (−0.07, 0.06)	0.75
Lumbar Spine T-Score	58	0.66 (0.21, 1.10)	61	0.53 (0.22, 0.84)	−0.13 (−0.66, 0.40)	0.78
Lumbar Spine Z-Score	58	−0.20 (−0.60, 0.20)	61	−0.14 (−0.42, 0.14)	0.06 (−0.42, 0.54)	0.47
	***N***	***No. (%)***	***N***	***No. (%)***	***Proportion (95% CI)***	
Total Hip Osteopenia	58	3 (5.2%)	62	14 (22.6%)	17.4% (5.5, 29.3)	0.007
Total Hip Osteoporosis	58	2 (3.4%)	62	1 (1.6%)	−1.8% (−7.4, 3.8)	0.64
Osteopenia (any area)	58	7 (12.1%)	62	18 (29.0%)	16.9% (2.8, 31.0)	0.03
Osteoporosis (any area)	58	3 (5.2%)	62	1 (1.6%)	−3.6% (−10.1, 2.9)	0.38

*Averaged across left and right sides.

† Characteristics are compared between HIV serostatus groups with Wilcoxon rank-sum test for BMD. Chi-square test of proportions for normal, osteopenia and osteoporosis between HIV serostatus groups are significant for hip (p = 0.02) and any area (p = 0.05) therefore results for pairwise tests of proportion (normal osteopenia and normal-osteoporosis) are reported.

The proportion of individuals with osteopenia (T-scores falling between −1 and −2.5) was significantly higher overall in HIV-infected individuals for all sites combined (P = 0.03), and specifically at the total hip (p = 0.007). The proportion of individuals with osteoporosis (T-scores of −2.5 or lower) however, was not significantly different for all sites combined nor for any specific site. Multiple logistic regression of factors associated with osteopenia or osteoporosis revealed a significant association (P = 0.02) with race (Caucasian vs. African American) in our study (Supplemental Table S1).

### Increased bone resorption and decreased bone formation in ART-naïve HIV-infected individuals

To assess the mechanics underlying the increase in osteopenia in HIV-infected individuals, we quantified the plasma concentrations of specific and sensitive markers of in vivo bone resorption (C-terminal telopeptide of collagen [CTx]) and bone formation (osteocalcin) (Table 3). Median CTx was 63% higher (P = 0.007) in ART-naïve HIV-infected individuals compared to controls. Conversely, the median concentrations of osteocalcin were 18% lower in HIV-infected individuals, although this difference fell just short of statistical significance (P = 0.06). After adjusting for baseline risk factors for low BMD including age, sex, race, BMI, smoking, alcohol consumption, and fracture history in multiple linear regression analyses, geometric mean plasma levels of both CTx and osteocalcin were statistically significantly different (P = 0.01 and P = 0.03 respectively) between HIV-infected and uninfected controls. Details of the multiple linear regressions are given in Supplemental Tables S1-S2. Although no additional significant associations were observed between CTx and other specific covariates (Supplemental Table S2), higher BMI values and alcohol consumption were associated with lower osteocalcin (P = 0.001 and P = 0.007 respectively) (Supplemental Table S3). This suggests that increased prevalence of osteopenia in HIV-infected individuals is related to a significant increase in bone resorption and likely compounded by a decline in bone formation. Low BMI and alcohol consumption may also directly contribute to diminished bone formation.

**Table 3:** Bone turnover outcomes.

**Outcome – Univariable Analysis** *	**N**	**HIV Status**	**Median (95% CI)**	**Difference**	**P**
CTx (ng/mL)	57	*HIV-*	0.19 (0.15, 0.28)	0.12 (0.02, 0.22)	0.007
	61	*HIV+*	0.31 (0.22, 0.38)		
Osteocalcin (ng/mL)	58	*HIV-*	6.0 (5.4, 7.2)	−1.2 (−2.5, 0.1)	0.06
	61	*HIV+*	4.9 (3.4, 5.3)		
**Outcome – Multivariable Analysis** †	**N**	**HIV Status**	**Adjusted Geometric Mean (95% CI)**	**Geometric Mean Ratio (95% CI)**	**P**
CTx (ng/mL)	56	*HIV-*	0.20 (0.15, 0.26)	1.51 (1.09, 2.10)	0.01
	61	*HIV+*	0.30 (0.23, 0.40)		
Osteocalcin (ng/mL)	57	*HIV-*	6.53 (5.14, 8.30)	0.72 (0.53, 0.97)	0.03
	61	*HIV+*	4.71 (3.66, 6.08)		

*Characteristics are compared between HIV serostatus groups with Wilcoxon rank-sum test for CTx and osteocalcin.

† Multivariable analysis adjusted for age, gender, race, BMI, smoking, past 30 day alcohol consumption and fracture history

### Increased B cell expression of RANKL and decreased B cell expression of OPG in ART-naïve HIV-infected individuals reflect changes in bone resorption

**Figure 1:**
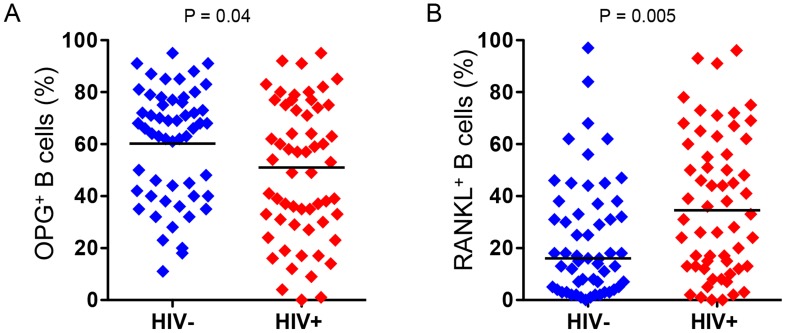
Increased frequency of RANKL-expressing B cells and decreased frequency of OPG-expressing B cells in HIV infection. Intracellular expression of: **A)** OPG and **B)** RANKL, by circulating peripheral blood B cells were quantified by flow cytometry in 56 HIV-negative and 57 HIV-infected individuals. Comparisons between HIV sero status were performed using Wilcoxon rank-sum test.

We [26] and others [19] have reported that B cells are a significant source of the bone protective factor OPG, but B cells can also be a significant source of the pro-resorptive cytokine RANKL [27], [28]. In fact, we recently reported that in HIV transgenic rats, B cell expression of OPG was significantly diminished, while B cell RANKL expression was significantly elevated [25]. We hypothesized that these disruptions to the immuno-skeletal interface may directly contribute to HIV-induced bone loss. To translate these animal findings into the human disease state, we quantified intracellular expression of OPG (Figure 1A) and RANKL (Figure 1B) in circulating peripheral blood B cells derived from HIV-infected and HIV-negative individuals. Univariable analysis (Table 4) revealed a 60% increase (P = 0.005), in the frequency of RANKL-expressing B cells and a 20% reduction (P = 0.04), in the frequency of OPG-expressing B cells in HIV-infected individuals compared to uninfected controls. In multivariable analyses (Table 4), means for both B cell RANKL and B cell OPG changes were significantly different between both groups (P = 0.01 and P = 0.03, respectively) after adjusting for the osteoporosis risk factors: age, sex, race, BMI, smoking, alcohol consumption and fracture history. Details of the multiple linear regressions are given in Supplemental Tables S2-S7.

**Table 4:** Summary statistics for B cell and plasma RANKL and OPG.

**Outcome – Univariable Analysis** *	**N**	**HIV Status**	**Median (95% CI)**	**Difference**	**P**
B cell OPG Expression (%)	56	*HIV-*	66.0 (60.0, 71.0)	−12.0 (−24.7, 0.7)	0.04
	57	*HIV+*	54.0 (37.0, 62.0)		
B cell RANKL Expression (%)	56	*HIV-*	16.5 (11.0, 29.0)	19.5 (3.9, 35.1)	0.005
	57	*HIV+*	36.0 (20.0, 48.0)		
Plasma Soluble OPG (pmol/L)	57	*HIV-*	2.8 (2.2, 3.0)	0.4 (−0.2, 1.0)	0.15
	61	*HIV+*	3.2 (2.6, 3.5)		
Plasma Soluble RANKL (pmol/L)	53	*HIV-*	0.18 (0.09, 0.38)	0.01 (−0.13, 0.15)	0.64
	47	*HIV+*	0.19 (0.09, 0.27)		
**Outcome – Multivariable Analysis** †	**N**	**HIV Status**	**Adjusted Mean (95% CI)**	**Mean Difference (95% CI)**	**P**
B cell OPG Expression (%)	55	*HIV-*	60.9 (53.3, 68.5)	−12.3 (−21.8, −2.8)	0.01
	57	*HIV+*	48.6 (40.5, 56.7)		
B cell RANKL Expression (%)	55	*HIV-*	21.6 (13.4, 29.9)	11.5 (1.2, 21.8)	0.03
	57	*HIV+*	33.1 (24.3, 41.9)		
Plasma Soluble OPG (pmol/L)	56	*HIV-*	2.55 (2.06, 3.04)	0.85 (0.27, 1.44)	0.005
	61	*HIV+*	3.40 (2.90, 3.91)		
Plasma Soluble RANKL (pmol/L)	52	*HIV-*	0.33 (0.22, 0.44)	−0.05 (−0.19, 0.09)	0.46
	47	*HIV+*	0.28 (0.16, 0.40)		

*Characteristics are compared between HIV serostatus groups with Wilcoxon rank-sum test.

† Multivariable analysis adjusted for adjusted for the baseline osteoporosis risk factors age, gender, race, BMI, smoking, past 30-day alcohol consumption and fracture history using multiple linear regression.

While B cell RANKL expression was not further associated with any covariates (Supplemental Table S4), a significant (P = 0.04) positive association between mean B cell OPG expression and alcohol consumption was revealed in multivariable analysis (Supplemental Table S5). The physiological importance of this observation, if any, is presently unclear.

These results validate our pre-clinical findings that B cell OPG expression declines in the context of HIV infection, while B cell RANKL expression increases. This imbalance in the immuno-skeletal interface may underlie or significantly contribute to the bone loss associated with human HIV infection.

### Changes in plasma soluble OPG and RANKL do not reflect changes in bone resorption

Because OPG and RANKL are considered key downstream effectors of osteoclast formation and bone resorption [17], [18], we quantified plasma concentrations of soluble total RANKL and OPG in HIV-infected and HIV-negative individuals. Despite significant increases in osteopenia and in the bone resorption marker CTx, median plasma soluble OPG and RANKL levels did not differ significantly between groups in univariable analyses (Table 4). In multivariable analysis (Table 4), after adjusting for the osteoporosis risk factors age, sex, race, BMI, smoking, alcohol consumption and fracture history, mean plasma RANKL was comparable between both groups, and did not differ with any other covariate, but a significant (P = 0.005) association was observed between adjusted mean plasma OPG concentrations and HIV serostatus. HIV infection was associated with increased, rather than diminished, plasma OPG concentration. Details of the multiple linear regressions are given Supplemental Tables S6-S7.

### B cell subset alterations in HIV infection

Majority of B cell defects in HIV infection with important consequences for pathogenesis are directly linked to the expansion or contraction of various B cell subsets, including significant loss of memory B cells, and expansion of activated and exhausted tissue-like memory B cells [29]. Median total B cell (CD20^+^) numbers were not significantly different between the HIV-negative (56 cells/µL, 95% CI 43, 70) and HIV-infected (62 cells/µL, 95% CI 44, 70) (P = 0.90) groups.

**Figure 2:**
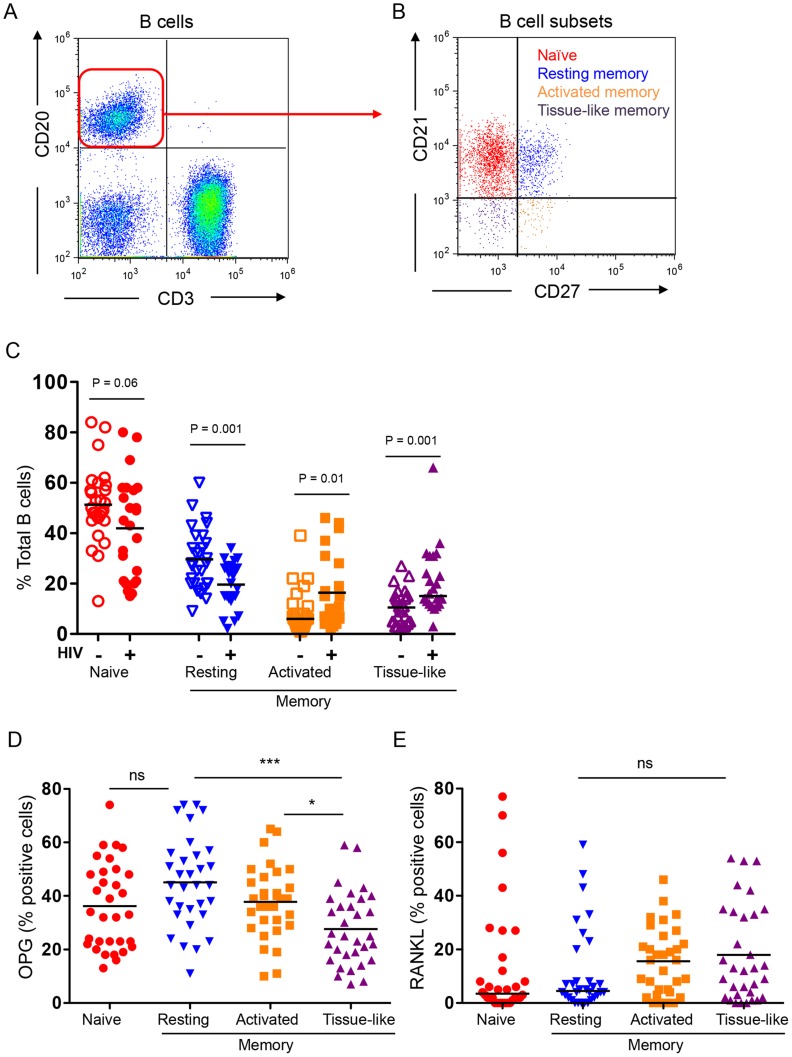
B cell subset RANKL and OPG expression in HIV-negative and HIV-positive individuals. **A**) Total B cells (CD3^−^CD20^+^ cells) were identified in the lymphocyte gate. **B**) B cells were further divided into 4 distinct subsets: naïve (CD21^hi^CD27^−^), resting memory (CD21^hi^CD27^+^), activated memory (CD21^−^CD27^+^), and exhausted tissue-like memory (CD21^−^CD27^−^). **C**) B cell subset distribution in HIV-negative (N = 28) and seropositive (N = 24) individuals. Intracellular expression of **D**) OPG and **E**) RANKL in B cell subsets of HIV-negative and seropositive individuals combined. Graphs reflect individual individuals with bars at the mean (parametric data) or median [non-parametric data, Activated and tissue-like memory subset (**C**) and naïve and resting memory (**E**)]. Simple comparisons were done using Student's *t* test (for parametric data) or Wilcoxon rank sum test (for non-parametric data) for each of the subsets (**C**) and one-way ANOVA was used to compare multiple groups (**D**). Actual P values are reported for simple comparisons. For ANOVA *P<0.05 or ***P<0.001 or P =  not significant (ns).

Using multi-color flow cytometry B cells (Figure 2A) were further segregated into naïve (CD21^hi^CD27^−^), resting memory (CD21^hi^CD27^+^), activated memory (CD21^−^CD27^+^), and exhausted tissue-like memory (CD21^−^CD27^−^) sub-populations (Figure 2B). HIV-infected individuals had dramatic alterations in B cell subsets (Figure 2C) including a significant decline in resting memory B cells (P = 0.0012) and significant expansion of activated memory and exhausted tissue-like memory B cells (P = 0.01 and P = 0.001 respectively). There was great variability in naïve B cell subsets within the HIV-infected population, with a net decrease in overall naïve B cells compared with HIV-negative controls, falling just short of statistical significance (P = 0.056).

### B cell subset OPG and RANKL expression

In order to determine which B cell subsets contribute RANKL and/or OPG, we quantified RANKL and OPG expression by intracellular staining and flow cytometry in a subset of uninfected (N = 17) and HIV-infected individuals (N = 15).

We found the highest and lowest frequencies of OPG-expressing cells in the resting memory and exhausted tissue-like memory B cells respectively (Figure 2D). By contrast RANKL expression was comparable in all four subsets, with higher frequencies of RANKL-expressing cells in the activated and exhausted tissue-like subsets (Figure 2E).

**Figure 3:**
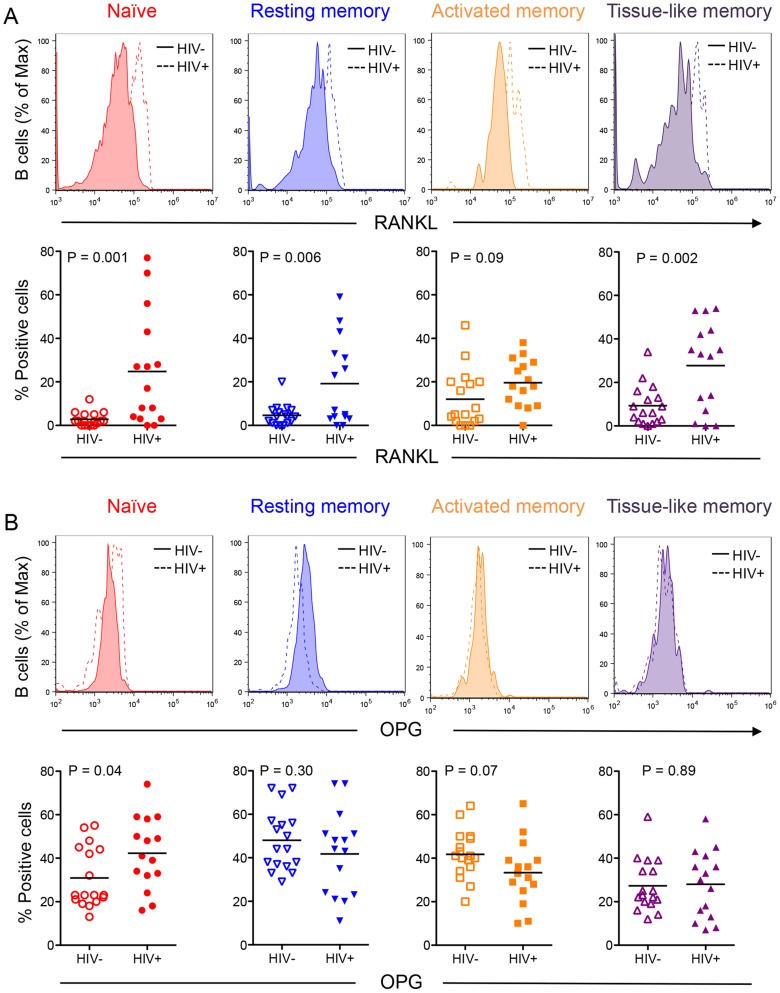
Differential B cell subset RANKL and OPG expression in HIV-negative and HIV-positive individuals. Representative histograms (alternate top panels) of B cell subset staining for RANKL (**A**) and OPG (**B**) for HIV negative individuals (solid line) and HIV positive individuals (dashed line). Scatter plots (alternate bottom panels) represent cumulative data for B cell subset RANKL (**A**) or OPG (**B**) expression from individual HIV-negative (HIV^−^, N = 17) or seropositive (HIV^+^, N = 15) individuals with bars at the mean (parametric data) or median [non-parametric data, RANKL: tissue-like memory (HIV-); OPG: All HIV- subsets]. Comparisons were done using two-way ANOVA, followed by pairwise comparisons of individual B cell subsets using student's t-test (parametric data) or Wilcoxon rank sum test (non-parametric data).

To examine how B cell subset OPG and RANKL expression is affected by HIV infection, we compared B cell subsets from uninfected and HIV-infected and HIV-negative subjects. Higher proportions of RANKL-expressing cells were observed in all four subsets in the HIV-positive compared to the HIV-negative. These differences were statistically significant for all but the activated memory B cell subset (Figure 3A). These data suggest that the increased expression of RANKL by total B cells (Figure 1B) is likely the consequence of a significant increase in RANKL-expressing B cell subpopulations (particularly activated and exhausted tissue-like memory B cells) in the context of HIV infection.

Despite an overall decrease in total B cell OPG (Figure 1A), a higher proportion of naive B cells from HIV-infected individuals produced OPG compared to controls (Figure 3B). Although a lower proportion of activated and exhausted tissue-like memory B cells from the HIV-positive group expressed OPG, this difference did not reach statistical significance.

### B cell RANKL/OPG ratio inversely correlates with BMD and T- and/or Z-scores at total hip and femoral neck, but no lumbar spine

In order to better understand our observed changes in B cell OPG and RANKL, we performed correlation analyses, with BMD (g/cm^2^, T-score and Z-score) from hip, femur neck and lumbar spine as outcomes and B cell OPG, RANKL, RANKL/OPG ratio and the natural log of this ratio as predictors, using the spearman rank correlation (Table 5). We analyzed both HIV- and HIV+ as a combined group (Table 5, Overall), and individually (Table 5, HIV-Negative and HIV-Positive). When analyzed together, we found a significant positive correlation between B cell OPG and femur neck Z-score, and falling just short of significance, for overall hip Z-score (p = 0.08). A negative correlation between RANKL/OPG ratio and hip Z-score and femur neck Z-score was also revealed. All measures of BMD (density, T-score and Z-score) for the hip and femur neck also negatively correlated with the log RANKL/OPG ratio. No significant correlations were observed in the HIV negative group alone. However, in the HIV positive group, B cell OPG was significantly correlated to femur neck Z-score and fell just short of significance for hip density and Z-scores (P = 0.06 for each). RANKL/OPG ratio was negatively correlated to hip density femur neck density and Z-scores. The log RANKL/OPG ratio was negative correlated to all BMD measures and T- and Z-scores at the hip and femur neck. No significant correlations between CTx and B cell RANKL or OPG were identified. Scatter plots of these correlation data for BMD (density, T-scores and Z-scores) versus B cell OPG, RANKL, RANKL/OPG ratio and RANKL/OPG ratio for HIV+ and HIV- individuals are also shown in supplemental Figures S2, S3 and S4 respectively.

**Table 5:** Association of bone mineral density and CTx with B cell OPG expression (%), B cell RANKL expression (%), RANKL/OPG ratio and Log RANKL/OPG ratio.

**Overall**	**B cell OPG**		**B cell RANKL**		**RANKL/OPG Ratio**		**Log RANKL/OPG Ratio**	
Outcome	r_s_	P	r_s_	P	r_s_	P	r_s_	P
**Hip density (g/cm^2^)**	0.140	0.14	−0.083	0.38	−0.144	0.13	**0.221**	**0.02**
**Hip T-score**	0.093	0.33	−0.105	0.27	−0.147	0.12	**−0.214**	**0.03**
**Hip Z-score**	0.166	0.08	−0.141	0.14	**−0.209**	**0.03**	**−0.262**	**0.006**
**Femur neck density (g/cm^2^)**	0.144	0.13	−0.082	0.39	−0.121	0.20	**−0.188**	**0.05**
**Femur neck T-score**	0.113	0.23	−0.115	0.23	−0.138	0.15	**−0.194**	**0.04**
**Femur neck Z-score**	**0.217**	**0.02**	−0.139	0.14	**−0.192**	**0.04**	**−0.224**	**0.02**
**Lumbar spine density (g/cm^2^)**	−0.123	0.20	0.040	0.68	0.071	0.46	0.020	0.84
**Lumbar spine T-score**	−0.130	0.17	0.024	0.80	0.056	0.56	0.002	0.98
**Lumbar spine Z-score**	−0.094	0.32	0.040	0.67	0.066	0.49	0.033	0.74
**CTx**	0.011	0.91	0.169	0.07	0.143	0.13	0.138	0.15
**HIV Negative**	**B cell OPG**		**B cell RANKL**		**RANKL/OPG Ratio**		**Log RANKL/OPG Ratio**	
Outcome	r_s_	P	r_s_	P	r_s_	P	r_s_	P
**Hip density (g/cm^2^)**	−0.033	0.81	0.010	0.94	0.038	0.79	−0.009	0.95
**Hip T-score**	−0.099	0.487	0.027	0.84	0.061	0.66	0.024	0.86
**Hip Z-score**	0.002	0.99	−0.068	0.62	−0.048	0.73	−0.084	0.54
**Femur neck density (g/cm^2^)**	0.042	0.76	0.075	0.58	0.082	0.55	0.032	0.82
**Femur neck T-score**	−0.005	0.97	0.084	0.54	0.096	0.48	0.050	0.71
**Femur neck Z-score**	0.105	0.44	0.018	0.89	0.006	0.97	−0.035	0.80
**Lumbar spine density (g/cm^2^)**	−0.138	0.31	0.053	0.70	0.077	0.57	0.032	0.81
**Lumbar spine T-score**	−0.189	0.17	0.044	0.75	0.081	0.55	0.037	0.79
**Lumbar spine Z-score**	−0.016	0.90	0.03	0.83	0.028	0.84	−0.021	0.88
**CTx**	−0.112	0.41	0.059	0.67	0.676	0.62	0.064	0.64
**HIV Positive**	**B cell OPG**		**B cell RANKL**		**RANKL/OPG Ratio**		**Log RANKL/OPG Ratio**	
Outcome	r_s_	P	r_s_	P	r_s_	P	r_s_	P
**Hip density (g/cm^2^)**	0.250	0.06	−0.138	0.30	**−0.278**	**0.04**	**−0.393**	**0.003**
**Hip T-score**	0.167	0.21	−0.162	0.23	−0.227	0.09	**−0.324**	**0.02**
**Hip Z-score**	0.246	0.06	−0.135	0.32	**−0.268**	**0.05**	**−0.336**	**0.01**
**Femur neck density (g/cm^2^)**	0.195	0.15	−0.202	0.13	**−0.272**	**0.04**	**−0.355**	**0.008**
**Femur neck T-score**	0.161	0.23	−0.245	0.07	−0.254	0.06	**−0.309**	**0.02**
**Femur neck Z-score**	**0.259**	**0.05**	−0.220	0.10	**−0.302**	**0.02**	**−0.325**	**0.02**
**Lumbar spine density (g/cm^2^)**	−0.078	0.57	−0.056	0.68	0.007	0.96	−0.059	0.68
**Lumbar spine T-score**	−0.085	0.53	−0.033	0.81	0.036	0.79	−0.030	0.83
**Lumbar spine Z-score**	−0.131	0.33	0.015	0.91	0.087	0.53	0.065	0.64
**CTx**	0.226	0.09	0.108	0.43	−0.056	0.69	−0.078	0.58

r_s_  =  Spearman rank correlation coefficient. Significant outcomes are in bold.

## Discussion

We recently documented severe skeletal deterioration resulting from elevated osteoclastic bone resorption, associated with disrupted B cell RANKL and OPG expression in the HIV transgenic rat model [25]. In the present study, we have translated these animal findings into humans by examining expression of RANKL and OPG by circulating B cells in HIV-infected individuals compared to HIV-negative controls. To limit life-style confounders we selected an “at risk for HIV infection” HIV-negative population with similar high rates of smoking and alcohol consumption as the HIV-infected group.

As we previously described in the HIV transgenic rat, here we observed a significant inversion in expression of RANKL and OPG by human peripheral blood B cells in HIV-infected individuals, demonstrating a potential immunocentric basis for bone loss in HIV infection. Detailed B cell subset analyses suggested that increased B cell RANKL was the result of all B cell subsets contributing more RANKL in the context of HIV infection, while the significant decrease in total B cell OPG is likely the net result of multiple changes in the distribution of B cell subsets. This includes a decrease in the proportion of resting memory B cells (a population that contains a high frequency of OPG-expressing cells) concurrent with a significant increase in exhausted tissue-like B cells (the population with the lowest frequency of OPG-expressing cells). Interestingly, a higher frequency of naïve B cells from HIV-infected individuals expressed OPG, an observation that likely reflects a previously described aberrant activated state of naïve B cells from HIV-infected individuals [30]. This may be a compensatory mechanism to make up for the loss of resting memory B cells, which contained the highest frequency of OPG-expressing cells.

Under conditions of normal physiological bone remodeling, bone homeostasis is regulated by a coordinated action of osteoclasts and osteoblasts and changes in the rate of bone resorption are balanced by changes in bone formation, a process referred to as coupling [31]. Interestingly, we found that increases in bone resorption in HIV-infected individuals were not counteracted by equivalent increases in bone formation, which actually demonstrated a significant decrease. This failure of osteoblastic bone formation to compensate for elevated resorption reveals an uncoupling of normal homeostatic mechanisms and likely contributes to the magnitude of bone loss in the HIV-infected individuals. Although the cause of uncoupling remains unknown, multivariable analysis (Supplemental Table S3) revealed that bone formation was significantly associated with alcohol consumption. Consistent with these findings serum levels of osteocalcin and histomorphometric analyses of bone formation have demonstrated that acute alcohol intoxication suppresses bone formation, but with either no effect, or increased effect, on bone resorption [32]. Excessive alcohol consumption has previously been associated with increased fracture risk in HIV-infected populations on ART [33]. Thus alcohol consumption in the face of HIV infection may further contribute to the overall magnitude of the decline in BMD by exacerbating the elevated rates of bone resorption.

BMI declines are commonly reported and associated with low BMD in HIV-infected individuals [10] and a significant decline in BMI was indeed evident in our HIV-infected group compared to HIV-negative controls. Importantly, in multivariable analyses, bone resorption, and B cell OPG and RANKL expression were not significantly associated with BMI, although BMI did correlate significantly with bone formation. Our data are consistent with a recent study by Cotter et al, reporting that HIV infection was independently associated with lower BMD at femoral neck, total hip and lumbar spine, respectively, after adjustment for demographic/lifestyle factors and BMI[34].

The effect of HIV infection on absolute BMD values fell short of statistical significance in our study, likely due to the predominantly African American male makeup of our local demographic, a population with characteristically high basal BMD. However, BMD T-scores, which are normalized for sex and race, were significantly increased overall and specifically at the hip in the HIV-infected group. Furthermore, significant differences in total hip Z-score, an index that further normalizes for subject age, also revealed significant skeletal decline in HIV-infected individuals relative to their uninfected peers. A further contributing factor to muted differences in BMD is the fact that we selected an “at risk” HIV-negative population, with high rates of smoking and alcohol consumption and other factors that may negatively impact basal BMD. Consequently, basal BMD in our control group may also be lower than the general population, reducing the apparent magnitude of the changes observed in turnover markers and BMD values in the seropositive group. In fact, modest negative Z-scores in the HIV-negative population may reflect this lower- than-expected basal BMD.

BMD itself is only a crude extrapolation of skeletal condition and does not take into account bone quality and structural properties that are important predictors of load bearing strength. Furthermore, DXA quantifies predominantly cortical bone, which comprises 80% of the skeleton. Changes in trabecular bone, which also contribute to load-bearing strength but represent only 20% of the total skeleton, are significantly underestimated by DXA. Because the trabecular compartment is more metabolically active than cortical bone, significant declines in trabecular bone mass may occur earlier than changes in cortical bone but may not be evident by DXA. This situation is further compounded by the relatively young age of our population (mean age  = 39 years) as BMD quantification and clinical definitions of osteopenia and osteoporosis involving DXA-based bone densitometry-derived T-scores do not correlate with fracture incidence in individuals under the age of ∼55 [35]. It has consequently been suggested that patient Z-scores may be more appropriately applied when assessing bone status in younger HIV-infected populations [36]. We did indeed observe that Z scores reflected more pronounced difference in BMD by HIV status than T scores.

We further quantified the relationship between the predictor variables: B cell OPG expression, B cell RANKL expression, B cell RANKL/OPG expression ratio, the natural log of RANKL/OPG ratio, and BMD measures (density, T-score and Z-score) and the bone turnover marker CTx. The RANKL/OPG ratio is considered to be an important index of bone resorption, and indeed we found significant correlations between the RANKL/OPG ratio with BMD and with T- and/or Z-scores in the long bones (femur neck and hip) of HIV-infected subjects. This association was however not evident in the HIV negative population, and is a likely consequence of the fact that RANKL production by B cells is not a feature of normal B cells in uninfected subjects. Interestingly there was no association between the RANKL/OPG ratio and the axial skeleton. As discussed above this may reflect the inadequacies of DXA based measurements to capture changes in cancellous bone rich sites such as spine. Although correlations are not evidence of cause effect relationships, taken together, our data support the concept that B cell alterations in RANKL and OPG production may contribute to decline of BMD in the context of HIV infection.

BMD-derived indices (density, T-scores and Z-scores) showed significant negative correlations with the B cell RANKL/OPG ratio at femoral neck and total hip, but not at the lumbar spine. Surprisingly, although the RANKL/OPG ratio is considered a final downstream effector of osteoclastogenesis and bone resorption, CTx, an index of resorption, did not significantly correlate with the B cell RANKL/OPG ratio. One possible explanation is that CTx is representative of global resorption across all bone surfaces in the body. Consequently, resorption in the lumbar spine, a metabolically active trabecular bone-rich site, may represent a relatively higher fraction of the total CTx pool, which may lead to an underrepresentation of other sites such as the hip and femoral neck. In fact, unlike BMD, CTx is not used clinically as a diagnostic tool, due in part to such limitations.

A limitation of our study is the fact that the patients served at our clinic are predominantly African American and males, and so our data may not be generalizable to other ethnic groups or females. Nonetheless, because HIV-associated bone loss has been documented in all racial groups and in both sexes, the same underlying mechanisms are likely involved.

Surprisingly, despite significant reductions in B cell OPG expression, serum OPG was observed to be modestly (∼9%) but significantly elevated, while RANKL was modestly diminished (−10%) in HIV-infected individuals, although the latter did not reach statistical significance. Our finding of elevated plasma OPG is consistent with previous studies in ART-naïve HIV-infected individuals relative to healthy controls [37]–[39], although Gibellini *et. al.*, reported elevated RANKL in their study as well [37]. Hwang *et. al.*, by contrast, identified diminished serum RANKL but did not find a significant change in circulating OPG [40].

Elevated OPG concentrations have been previously associated with increased BMD and Z-scores in ART-naive individuals [41] an overall increase in OPG, such as we observed in our studies, may be beneficial in resisting bone breakdown in our HIV individuals. However, the findings of increased OPG are not consistent with the overall elevated bone resorption observed by our group and others in HIV-infected individuals. These changes in circulating factors may thus not truly reflect RANKL and OPG concentrations in the bone microenvironment and consequently circulating factors may provide an inaccurate picture of bone metabolic status at local sites in organs and tissues.

Another limitation of our study was that we were restricted to circulating peripheral blood B cells that may not truly reflect the true magnitude of changes in B cells within the bone marrow. Nonetheless, we were able to discern significant changes in cellular expression of RANKL and OPG that were consistent with the changes observed in bone resorption. These changes were also consistent with bone marrow and splenic B cell changes observed in the HIV transgenic rat. Because B-lineage cells constitute a major bone marrow population (∼25%) as well as a dominant source of OPG and of RANKL under basal conditions and inflammatory/pathological conditions respectively, bone marrow-resident B cells likely have a very significant effect on basal bone homeostasis. In fact, we have previously reported that B cell knockout mice have significantly increased basal osteoclastic bone resorption and diminished skeletal mass, as a consequence of diminished bone marrow OPG. Bone loss was rescued in these B cell deficient animals by reconstitution of B cells [26]. Taken together, our data suggest that expression of RANKL and OPG by circulating B cells may better reflect skeletal status than circulating concentrations of these same factors.

**Figure 4:**
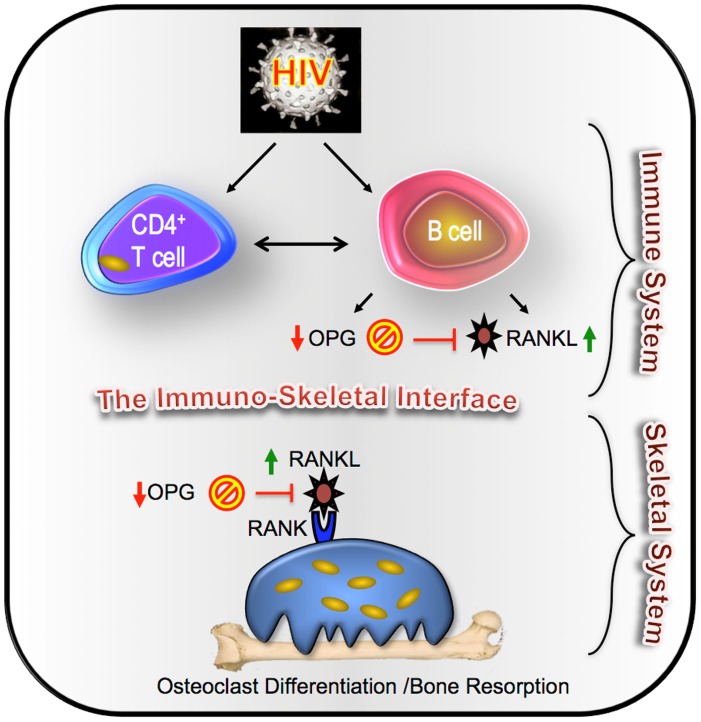
Immunocentric model of HIV induced bone loss. We propose a model for HIV- induced bone loss whereby HIV infection, either through direct effects on B cells, or though disruption of T cell costimulation of the humoral system, leads to a decline in the frequency of B cells secreting OPG, coupled with an increase in the frequency of B cells secreting RANKL. This disruption of the immuno-skeletal interface results in an increase in the RANKL/OPG ratio that is permissive for osteoclast formation and enhanced bone resorption, which ultimately contributes to bone loss and the elevated bone fracture risk characteristic of HIV-infected individuals.

In conclusion, our data confirm a significant imbalance in circulating B cell RANKL and OPG expression consistent with enhanced osteoclastic bone resorption and establish a potential immunocentric basis for bone loss (summarized diagrammatically in Figure 4). This imbalance in the immuno-skeletal interface may ultimately contribute to higher rates of fracture incidence in HIV-infected individuals.

## Materials and Methods

### Study design and population

This was a cross-sectional study conducted in HIV-negative and antiretroviral-naïve HIV-infected male and female adult volunteers. All individuals were recruited from an urban outpatient HIV clinic (the Grady Infectious Diseases Program, Atlanta, Georgia, USA) between November 2010 and December 2012. At enrollment, demographic information, medication history, smoking history and family history of fracture after 18 years of age, as well as clinical and laboratory data were collected (Table 1). Additional eligibility criteria included age ≥30 ≤50 years and absence of active malignancy, pathologic bone disease, or other immunologic conditions. Of note, BMD peaks around 25 years of age and then declines progressively. Significant alterations to bone turnover and structure ensue following menopause in women and with gonadal decline with advancing age in men. Restricting the age of study participants thus reduced the confounding effects of age on bone turnover and mass. Exclusion criteria included: individuals on any medication with known effects on the immune and/or skeletal systems (e.g. immune modulation therapy, systemic glucocorticoids, steroid hormones, bisphosphonates or other anti-resorptive agents or Teriparatide) within the preceding 8 weeks, were not fully ambulatory, had significant renal and/or hepatic impairment, or were pregnant or breastfeeding.

This study was registered at ClinicalTrials. Gov under the title “Effect of HIV Infection and Highly Active Antiretroviral Treatment (HAART) on Bone Homeostasis (OPG-2)” and was assigned the ClinicalTrials.gov Identifier: NCT01020045.

### Ethics statement

All individuals provided written informed consent before undergoing any study procedures, and the study was designed according to the WHO ethical guidelines for human studies and approved by the Institutional Review Board of Emory University.

### Study procedures and clinical assays

At enrollment, demographic information, detailed medication history, clinical and laboratory data, including complete blood counts with platelets and differential and full chemistry profiles, were collected.

### Plasma viremia and chemistries

HIV sero-status was confirmed as negative in healthy volunteers by rapid enzyme-linked immunosorbent assay (ELISA) test after pre-test counseling. In HIV-seropositive individuals, documentation of infection by ELISA test, western blot and/or plasma HIV RNA was recorded. Baseline plasma HIV RNA was measured with the Abbott Real Time HIV PCR assay (Abbott Molecular, Des Plaines, IL, USA) and CD4^+^ T cell counts with percentage were obtained for the HIV-seropositive group.

### Bone densitometry by Dual energy x-ray absorptiometry (DXA)

Left and right total hip BMD was assessed using the same dual energy x-ray absorptiometry (DXA) machine (Lunar prodigy scanner [GE Lunar, Madison, WI.]) and the same software (Encore Software, v.2010 13.31) for all participants at Emory University Hospital. Rates of osteoporosis and osteopenia were assessed using T-scores derived by comparing the subject's BMD to that of a reference database reflecting peak BMD values, adjusted for gender and ethnicity. Osteopenia was defined as T-scores between −1.0 and −2.5 and osteoporosis as T-scores of −2.5 or lower, per WHO criteria [42]. We further computed Z-scores, comparing subject BMD to an age matched reference database adjusted for gender and ethnicity.

### Cell and plasma collection and storage

Peripheral blood mononuclear cells (PBMC) and plasma were isolated from cell processing tubes (CPT, BD Vacutainer) after density gradient centrifugation. Plasma was stored at −80°C and PBMCs were resuspended in freezing medium (10% DMSO + individual participants' plasma) and stored in liquid N_2_ (−181°C) until use. Lymphocytes from HIV-negative and HIV-infected individuals were found to be between 95%–97% and 90%–93% viable respectively, upon thawing. After 18 hr at 37°C, viability of PBMC from HIV-negative and infected individuals declined to 93% and 78% respectively. Dead cells were excluded from all flow cytometry analyses by gating.

### Flow cytometry

#### Direct surface staining and intracellular staining

The following anti-human monoclonal antibodies were used to stain for total B cells and subsets: CD3 (clone HIT3a), CD20 (clone 2H7), CD21 (clone M-T271), and CD27 (clone 2H7).

#### Intracellular staining for spontaneous B cell OPG and RANKL expression

Thawed PBMC were rested overnight at 37°C, and cells were incubated with a protein transport inhibitor containing monensin (Golgistop, BD Biosciences) for the last 5 hours of the 16- to 18-hour recovery period. For intracellular staining cells were first stained for surface markers and then fixed and permeabilized using the BD Cytofix/Cytoperm Fixation/permeabliziation kit (BD Biosciences). To quantify intracellular OPG, cells were incubated with 5 µg/ml of anti-human OPG-biotin (Leinco Technologies) for 30 minutes at 4°C, followed by incubation with Streptavidin-PE. RANKL was quantified by incubation with 5 µg/ml recombinant OPG-Fc (R&D systems) for 30 minutes at 4°C as previously described [43], followed by anti-human IgG Fc-PE (BD Biosciences) for another 30 minutes at 4°C. Gates were set as shown in Supplemental Figure S1, using fluorescence-minus-one (FMO) controls to establish specific binding ranges. All cells were fixed in 4% paraformaldehyde and analyzed on an Accuri flow cytometer (BD Immunocytometry Systems, San Jose, CA). Data were analyzed using FlowJo software version 9.6 (Treestar, San Carlos CA).

### Enzyme-linked immunosorbent assay (ELISA)

Commercial ELISAs were used to measure plasma CTx and osteocalcin (Immunodiagnostic Systems, Scottsdale, AZ), and OPG and total soluble RANKL (Alpco Diagnostics, Salem, NH) according to the manufacturers' instructions.

### Statistical analyses

An extensive detailed report of the statistical analysis is located in the Supplemental Methods S1. Prior to implementation of any specific statistical analysis, all assumptions were assessed. For instance, the appropriateness of the assumptions of normality and homogeneity of variance were examined. If found to be violated, alternative methods such as data transformations or non-parametric procedures were used. We used two-sample Student's *t* test for parametric data, or Wilcoxon rank-sum test for nonparametric data, to compare differences between HIV-negative and positive groups in univariable analyses. Because a number of factors can impact bone density and bone turnover the data was further adjusted for 7 major covariates: enrollment age, gender, race, BMI, current smoking status, past 30 day alcohol use and history of bone fracture (after 18 years of age) as potential predictors of select bone biomarkers and BMD using multiple linear regression. A natural logarithm transformation was performed on the biomarkers CTx and osteocalcin prior to regression analysis to help improve symmetry in the distribution and to help ensure an appropriate model fit based on regression residual plots. Pearson's product moment correlation method was used for correlations. The multivariable results are summarized with adjusted means and mean differences for non-transformed variables and adjusted geometric means and geometric mean ratios for log-transformed variables, along with 95% CIs. The adjusted mean for each subgroup (HIV-infected or HIV-negative) was defined as the mean response obtained by fitting the statistical model at the mean age and the mean BMI of the two subgroups and averaged across levels of the other risk factors.

Prevalence of osteopenia (BMD T-score between -1.0 and -2.5) or osteoporosis (BMD T-score of -2.5 or lower) was summarized by group with proportion and proportion difference reported with 95% CIs and compared with a Chi-square test. Statistical analyses were performed using SAS software (Cary, NC).

B cell subset analyses were performed using GraphPad Prism for Mac OS X software (La Jolla, CA). Prior to the statistical analyses, normal distribution and homogeneity of the variances were tested. To analyze the differences in subset distribution between HIV-negative and HIV-infected groups, simple comparisons were done using Student's *t* test (for parametric data) or Wilcoxon rank sum test (for non-parametric data) for each of the subsets.

For B cell subset expression of OPG and RANKL, first a one-way analysis of variance (ANOVA) was used to compare the mean intracellular expression of OPG by type of B cell subset (naïve; resting, activated and tissue-like memory) regardless of HIV status. If an overall difference was identified, a Bonferroni adjustment was used to adjust for multiple comparisons. Secondly, HIV status and B cell subset were used as predictors in two-way ANOVA, followed by pairwise comparisons of individual B cell subsets using student's t-test (parametric data) or Wilcoxon rank sum test (non-parametric data). All statistical tests were 2-sided and P-values ≤0.05 were considered statistically significant.

### Correlation analyses

We quantified the relationships between B cell OPG and RANKL, with the bone resorption marker CTx and with BMD (density, T-score and Z-score) from hip, femur neck and lumbar spine as outcomes and B cell OPG, RANKL, RANKL/OPG ratio and the natural log of RANKL/OPG ratio as predictors. The distribution of RANKL/OPG ratio was highly skewed but the natural log transformation of the ratio had an approximately normal distribution. Left/right measurements from hip and femur neck were averaged as before. We quantified the relationships between outcome and predictor variables with a non-parametric (Spearman's rank correlation) univariable method. Correlation analyses were performed using SAS software (Cary, NC).

## Supporting Information

Figure S1 **Gating scheme for OPG and RANKL staining of B cells by flow cytometry.** (A) Lymphocyte populations were preselected based on SSC and FSC gating and (**B**) resolved into B cells and T cells by CD20 and CD3 staining respectively. (**C**) B cells were further stained for intracellular OPG or (**D**) RANKL. Positive staining was assessed with the aid of isotype matched FMO controls (gray line) and gates set by reference to positive and negative staining queues based on differential staining by HIV positive and negative populations. Specifically, for OPG (**C**) HIV negative B cells revealed high expression patterns with large numbers of total B cells expressing OPG while B cells from HIV positive patients contained both OPG positive and negative populations. By contrast, the majority of HIV negative B cells did not express RANKL (**D**) while a high percentage of B cells from HIV positive subjects did.
(DOCX)

Figure S2 **Association of bone mineral density (BMD) with B cell OPG expression (%) in HIV-negative and HIV-positive individuals.** HIV-negative subjects are shown in blue circles and HIV-positive in red circles. The Spearman rank sum test was used to assess the relationship between B cell OPG expression and BMD (density, g/cm^2^, T-score and Z-score) at the hip, femur neck and lumbar spine.
(DOCX)

Figure S3 **Association of bone mineral density (BMD, T-score and Z-score) with B cell RANKL production (%) in HIV-negative and HIV-positive individuals.** HIV-negative subjects are shown in blue circles and HIV-positive in red circles. The Spearman rank sum test was used to assess the relationship between B cell RANKL expression and BMD (density, g/cm^2^, T-score and Z-score) of the hip, femur neck and lumbar spine.
(DOCX)

Figure S4 **Association of bone mineral density (BMD, T-score and Z-score) with the B cell RANKL/OPG production ratio in HIV-negative and HIV-positive individuals.** HIV-negative subjects are shown in blue circles and HIV-positive in red circles. The Spearman rank sum test was used to assess the relationship between B cell RANKL expression and BMD (density, g/cm^2^, T-score and Z-score) of the hip, femur neck and lumbar spine.
(DOCX)

Table S1 **Multiple logistic regression of race and BMI with any osteopenia or osteoporosis.**
(DOCX)

Table S2 **Multiple linear regression for CTx, adjusted for HIV status, age (continuous), gender, race, BMI (continuous), smoking, alcohol and fracture.** Age and BMI are used as continuous variables in the multiple linear regression model. Group adjusted geometric mean estimates for age and BMI shown in the table are taken from multifactor ANOVA model.
(DOCX)

Table S3 **Multiple linear regression for osteocalcin adjusted for HIV status, age (continuous), gender, race, BMI (continuous), smoking, alcohol and fracture.** Age and BMI are used as continuous variables in the multiple linear regression model. Group adjusted geometric mean estimates for age and BMI shown in the table are taken from multifactor ANOVA model.
(DOCX)

Table S4 **Multiple linear regression for B cell RANKL expression (%) adjusted for HIV status, age (continuous), gender, race, BMI (continuous), smoking, alcohol and fracture.** Age and BMI are used as continuous variables in the multiple linear regression model. Group mean estimates for age and BMI shown in the table are taken from multifactor ANOVA model.
(DOCX)

Table S5 **Multiple linear regression for B cell OPG production (%) adjusted for HIV status, age (continuous), gender, race, BMI (continuous), smoking, alcohol and fracture.** Age and BMI are used as continuous variables in the multiple linear regression model. Group mean estimates for age and BMI shown in the table are taken from multifactor ANOVA model.
(DOCX)

Table S6 **Multiple linear regression for plasma soluble OPG (pmol/L) adjusted for HIV status, age (continuous), gender, race, BMI (continuous), smoking, alcohol and fracture.** Age and BMI are used as continuous variables in the multiple linear regression model. Group adjusted mean estimates for age and BMI shown in the table are taken from multifactor ANOVA model.
(DOCX)

Table S7 **Multiple linear regression for plasma soluble RANKL adjusted for HIV status, age (continuous), gender, race, BMI (continuous), smoking, alcohol and fracture.** Age and BMI are used as continuous variables in the multiple linear regression model. Group adjusted mean estimates for age and BMI shown in the table are taken from multifactor ANOVA model.
(DOCX)

Methods S1 **Detailed statistical analyses of all results.**
(DOCX)
