# Supplementary material for: Dysregulated B Cell Expression of RANKL and OPG Correlates with Loss of Bone Mineral Density in HIV Infection
Source: PLoS Pathog. 2014 Nov 13;10(11):e1004497. doi: 10.1371/journal.ppat.1004497 (PMC4231117; doi:10.1371/journal.ppat.1004497)
Supplement: Table S1 — Multiple logistic regression of race and BMI with any osteopenia or osteoporosis. (DOCX) [file ppat.1004497.s005.docx]

***Table S1: Multiple Logistic Regression of Factors Associated with Any Osteopenia or Osteoporosis, N=119***

| ***Variable (Effect)*** | ***Estimate*** | ***Standard Error*** | ***Odds Ratio*** | ***95% CI*** | ***P*** |
| --- | --- | --- | --- | --- | --- |
| Race (*Black vs White*) | -0.7331 | 0.3042 | 0.23 | 0.07-0.76 | **0.02** |
| BMI (*per 1 unit increase*) | -0.1545 | 0.0512 | 0.86 | 0.78-0.95 | **0.003** |
