# Supplementary material for: Dysregulated B Cell Expression of RANKL and OPG Correlates with Loss of Bone Mineral Density in HIV Infection
Source: PLoS Pathog. 2014 Nov 13;10(11):e1004497. doi: 10.1371/journal.ppat.1004497 (PMC4231117; doi:10.1371/journal.ppat.1004497)
Supplement: Table S6 — Multiple linear regression for plasma soluble OPG (pmol/L) adjusted for HIV status, age (continuous), gender, race, BMI (continuous), smoking, alcohol and fracture. Age and BMI are used as continuous variables in the multiple linear regression model. Group adjusted mean estimates for age and BMI shown in the table are taken from multifactor ANOVA model. (DOCX) [file ppat.1004497.s010.docx]

***Table S6: Multiple Linear Regression for Plasma Soluble OPG (pmol/L)***

***Adjusted for HIV status, Age (continuous), Gender, Race, BMI (continuous), Smoking, Alcohol and Fracture***

***N=117***

| ***Variable*** | ***Group*** | ***Adjusted Mean (95% CI)*** | ***P*** | |
| --- | --- | --- | --- | --- |
| HIV Status | *Negative* | 2.55 (2.06-3.04) | **0.005** | |
|  | *Positive* | 3.40 (2.90-3.91) |  |  |
| Age* | *30-40 years old* | 2.90 (2.37-3.43) | 0.16 | |
|  | *40-50 years old* | 3.13 (2.67-3.59) |  |  |
| Sex | *Female* | 3.40 (2.85-3.96) | **0.01** | |
|  | *Male* | 2.55 (2.08-3.03) |  |  |
| Race | *Black* | 2.99 (2.65-3.32) | 0.95 | |
|  | *White* | 2.97 (2.24-3.70) |  |  |
| BMI (quartiles)* | *Q1 (<22.7)* | 3.05 (2.37-3.72) | 0.33 | |
|  | *Q2 (22.7-25.9)* | 2.83 (2.16-3.50) |  |  |
|  | *Q3 (25.9-32.4)* | 3.08 (2.48-3.68) |  |  |
|  | *Q4 (>32.4)* | 3.09 (2.42-3.76) |  |  |
| Current smoking | *No* | 2.77 (2.26-3.28) | 0.15 | |
|  | *Yes* | 3.18 (2.72-3.65) |  |  |
| Past 30 day Alcohol use | *No* | 2.83 (2.32-3.33) | 0.28 | |
|  | *Yes* | 3.13 (2.66-3.60) |  |  |
| History of bone fracture  (after 18 years of age) | *No* | 2.85 (2.38-3.32) | 0.41 | |
|  | *Yes* | 3.11 (2.56-3.66) |  |  |
| **Age and BMI are used as continuous variables in the multiple linear regression model. Group adjusted mean estimates for age and BMI shown in the table are taken from multifactor ANOVA model.* | | | |  |
