# Supplementary material for: Dysregulated B Cell Expression of RANKL and OPG Correlates with Loss of Bone Mineral Density in HIV Infection
Source: PLoS Pathog. 2014 Nov 13;10(11):e1004497. doi: 10.1371/journal.ppat.1004497 (PMC4231117; doi:10.1371/journal.ppat.1004497)
Supplement: Methods S1 — Detailed statistical analyses of all results. (DOCX) [file ppat.1004497.s012.docx]

**Supplemental Methods, S1:**

***Detailed Statistical Analyses***:

Demographic and clinical characteristics are compared between the HIV-negative and treatment naïve HIV-infected groups with the two-sample t-test for continuous variables and with a Chi-square test for proportions (Table 1). Bone biomarkers, BMD, and osteopenia and osteoporosis are summarized by and compared between HIV groups (Tables 2 and 3).

Nonparametric statistics and methods are used for bone biomarkers (median and median difference with 95% confidence intervals and Wilcoxon rank sum test) and parametric statistics and methods are used for BMD (mean and mean difference with 95% confidence intervals and two-sample t-test). The choice of parametric versus nonparametric methods was driven by normality versus non-normality of data distributions.

BMD was expressed as a density, a T-score, and a Z-score. T- and Z-scores represent the differences in standard deviations from mean reference BMD values from the general population matched for race and sex (T-score) and matched for race, sex and age (Z-score). A Z-score of zero represents a BMD measurement that is the normal mean value for the patient based on their race, sex and age. Prevalence of osteopenia (BMD T-score between 1 and -2.5 or osteoporosis (BMD T-score ≤ -2.5) are summarized by group with proportion and proportion difference reported with 95% confidence intervals and tested with a Chi-square test. The Spearman rank correlation coefficient is used to determine the association between age and BMD outcomes (Table 2), and age and bone biomarkers (Table 3), for HIV negative and positive groups.

A covariate-adjusted analysis was performed including HIV status, enrollment age, gender, race, BMI, current smoking status, any past 30 day alcohol use and history of bone fracture (after 18 years of age) as potential predictors of select bone biomarkers and BMD in multiple linear regression (SAS PROC GLM; Table 4).

A natural logarithm transformation was performed on biomarkers CTx and osteocalcin prior to regression analysis to help improve symmetry in the distribution and to help ensure an appropriate model fit based on regression residual plots. Biomarker measurements that were below the lower limit of detection (LLD) were set to half of the LLD prior to log transformation. Several biomarkers had values reported below their LLD including CTx (3 values below 0.02 ng/mL), osteocalcin (1 value below 0.50 ng/mL), Plasma Soluble OPG (3 values below 0.14 pmol/L) and Plasma Soluble RANKL (7 values below 0.02 pmol/L).

The multivariable results are summarized with adjusted means and mean differences for non-transformed variables and adjusted geometric means and geometric mean ratios for log-transformed variables, along with 95% confidence intervals.

The adjusted mean for each subgroup (HIV positive or negative) is defined as the mean response obtained by fitting the statistical model at the mean age and the mean BMI of the two subgroups and averaged across levels of the other risk factors. Adjusted means and 95% confidence intervals for each outcome were calculated for each risk factor in the presence of others in the final model; age at enrollment and body mass index were included as continuous covariates in the regression models and summarized as categorical covariates (Supplementary Tables 1-6).

Covariate adjusted analyses were also performed with any osteopenia or osteoporosis as the outcome in multiple logistic regression (SAS PROC LOGISTIC). However, due to low prevalence (29 events), the multivariable model was built using a backwards-stepwise selection to choose prognostic variables, with HIV status retained as a covariate regardless of significance. The odds ratio and its 95% confidence interval were calculated for each factor in the presence of others in the final model.

All statistical tests are 2-sided. A P-value ≤ 0.05 is considered statistically significant.
