# Supplementary material for: Dysregulated B Cell Expression of RANKL and OPG Correlates with Loss of Bone Mineral Density in HIV Infection
Source: PLoS Pathog. 2014 Nov 13;10(11):e1004497. doi: 10.1371/journal.ppat.1004497 (PMC4231117; doi:10.1371/journal.ppat.1004497)
Supplement: Supplementary file 9 [file ppat.1004497.s001.docx]

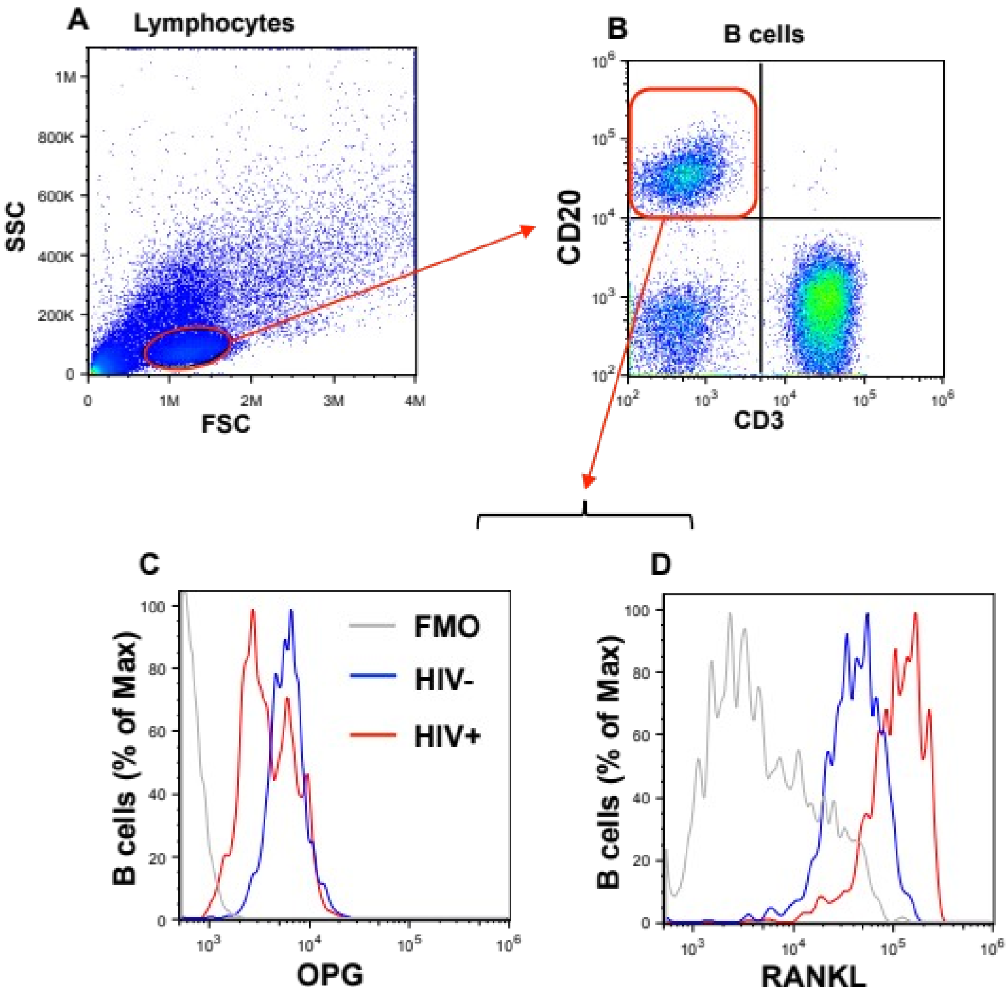

Figure S1: Gating scheme for OPG and RANKL staining of B cells by flow cytometry. (**A**) Lymphocyte populations were preselected based on SSC and FSC gating and (**B**) resolved into B cells and T cells by CD20 and CD3 staining respectively. **(C)** B cells were further stained for intracellular OPG or (**D**) RANKL. Positive staining was assessed with the aid of isotype matched FMO controls (gray line) and gates set by reference to positive and negative staining queues based on differential staining by HIV positive and negative populations. Specifically, for OPG (**C**) HIV negative B cells revealed high expression patterns with large numbers of total B cells expressing OPG while B cells from HIV positive patients contained both OPG positive and negative populations. By contrast, the majority of HIV negative B cells did not express RANKL (**D**) while a high percentage of B cells from HIV positive subjects did.
