# Supplementary material for: Dysregulated B Cell Expression of RANKL and OPG Correlates with Loss of Bone Mineral Density in HIV Infection
Source: PLoS Pathog. 2014 Nov 13;10(11):e1004497. doi: 10.1371/journal.ppat.1004497 (PMC4231117; doi:10.1371/journal.ppat.1004497)
Supplement: Supplementary file 11 [file ppat.1004497.s003.docx]

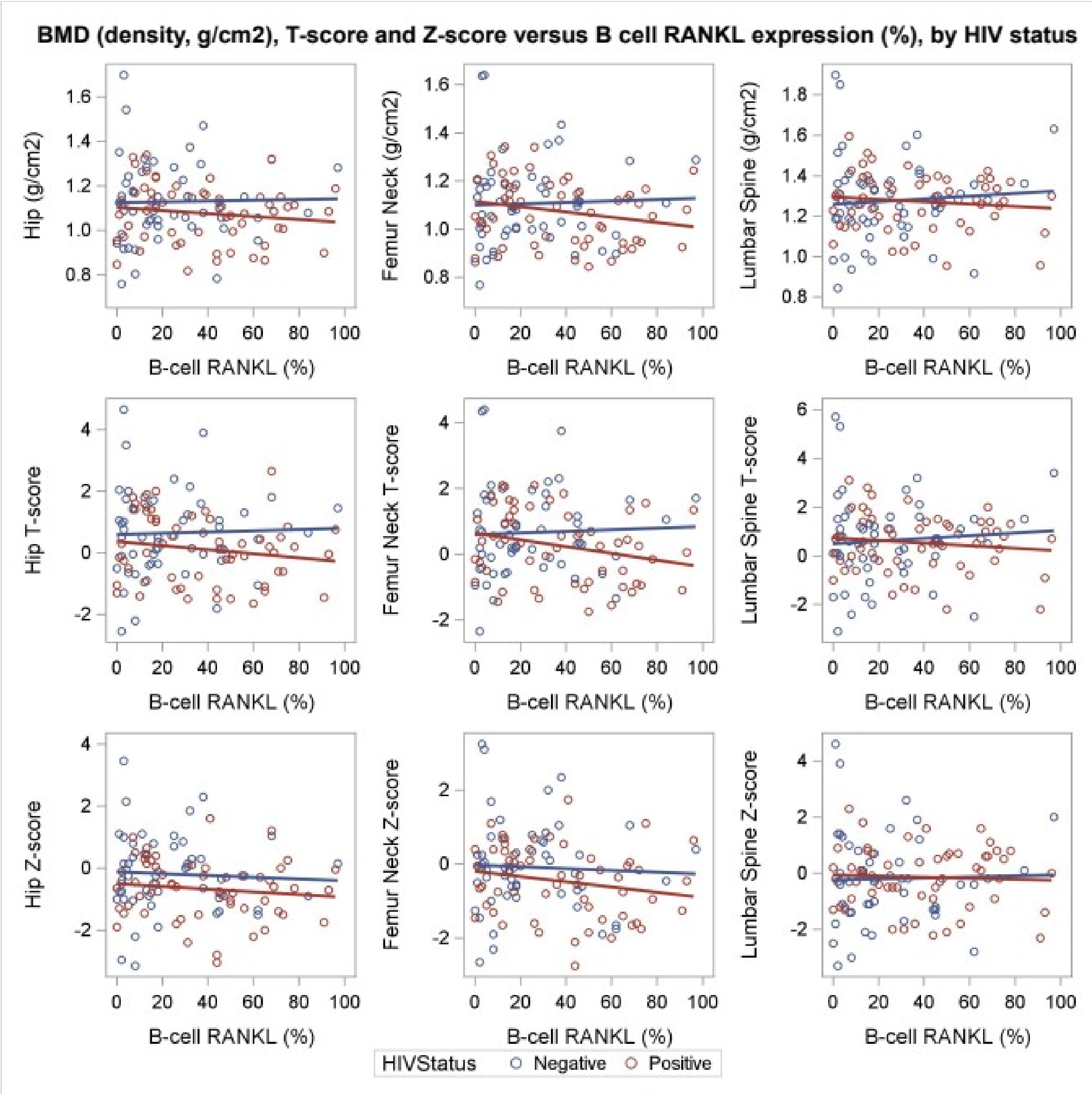

Figure S3: Association of bone mineral density (BMD, T-score and Z-score) with B cell RANKL production (%) in HIV-negative and HIV-positive individuals.

HIV-negative subjects are shown in blue circles and HIV-positive in red circles. The Spearman rank sum test was used to assess the relationship between B cell RANKL expression and BMD (density, g/cm^2, T-score and Z-score) of the hip, femur neck and lumbar spine.
